# Nanopore Sequencing Resolves Elusive Long Tandem-Repeat Regions in Mitochondrial Genomes

**DOI:** 10.3390/ijms22041811

**Published:** 2021-02-11

**Authors:** Liina Kinkar, Robin B. Gasser, Bonnie L. Webster, David Rollinson, D. Timothy J. Littlewood, Bill C.H. Chang, Andreas J. Stroehlein, Pasi K. Korhonen, Neil D. Young

**Affiliations:** 1 Department of Veterinary Biosciences, Melbourne Veterinary School, Faculty of Veterinary and Agricultural Sciences, The University of Melbourne, Parkville, Victoria 3010, Australia; liina.kinkar@unimelb.edu.au (L.K.); bill.chang@yourgene-health.com (B.C.H.C.); astroehlein@unimelb.edu.au (A.J.S.); pasi.korhonen@unimelb.edu.au (P.K.K.); 2 Department of Life Sciences, Natural History Museum, London SW7 5BD, UK; b.webster@nhm.ac.uk (B.L.W.); d.rollinson@nhm.ac.uk (D.R.); t.littlewood@nhm.ac.uk (D.T.J.L.); 3 London Centre for Neglected Tropical Disease Research, London W12 1PG, UK

**Keywords:** *Schistosoma haematobium*, mitochondrial (mt) genome, tandem-repetitive DNA, non-coding (control) region, Oxford Nanopore technology, informatics

## Abstract

Long non-coding, tandem-repetitive regions in mitochondrial (mt) genomes of many metazoans have been notoriously difficult to characterise accurately using conventional sequencing methods. Here, we show how the use of a third-generation (long-read) sequencing and informatic approach can overcome this problem. We employed Oxford Nanopore technology to sequence genomic DNAs from a pool of adult worms of the carcinogenic parasite, *Schistosoma haematobium*, and used an informatic workflow to define the complete mt non-coding region(s). Using long-read data of high coverage, we defined six dominant mt genomes of 33.4 kb to 22.6 kb. Although no variation was detected in the order or lengths of the protein-coding genes, there was marked length (18.5 kb to 7.6 kb) and structural variation in the non-coding region, raising questions about the evolution and function of what might be a control region that regulates mt transcription and/or replication. The discovery here of the largest tandem-repetitive, non-coding region (18.5 kb) in a metazoan organism also raises a question about the completeness of some of the mt genomes of animals reported to date, and stimulates further explorations using a Nanopore-informatic workflow.

## 1. Introduction

Mitochondrial (mt) genomes display marked diversity in size and sequence among eukaryotic lineages, ranging from 6 kb in *Plasmodium falciparum* (malaria parasite) to >11 Mb in *Silene conica* (catchfly plant) [1,2]. In contrast to plants, fungi and numerous protists, published evidence indicates that the mt genomes of most metazoans appear to be remarkably compact, with seemingly limited variation in size [3,4,5,6,7]. Most animal mt genomes, particularly those of bilaterians, are 15–20 kb in size and encode ~37 genes [3,4]. Non-coding regions are usually reported to be short, apart from a ‘control region’ which often contains tandem-repetitive elements, usually comprising no more than 1.5 kb of the mt genome [8]. However, some early studies [9,10,11] had suggested the presence of long tandem-repeat regions of many thousands of nucleotide bases in mt genomes, which have remained largely elusive due to the technological challenges associated with sequencing them.

Long stretches of repetitive DNA are notoriously difficult to sequence using conventional Sanger- and second-generation (short-read) sequencing methods [12]. Repetitive elements that extend beyond the usual read length capacity of these platforms (~1 kb for Sanger sequencing; 100–300 bp for second-generation methods) cannot be reliably assembled (e.g., [13]). Although long-range PCR can be used to amplify DNA regions of several kilobases, sequencing through repetitive regions often leads to erroneous and/or ambiguous sequence reads as a consequence of self-priming of randomly-amplified repeat-segments, chimeras and/or jumping PCR artefacts [14,15]. Third-generation, single-molecule sequencing platforms, such as ‘PacBio’ and ‘Oxford Nanopore’, which can achieve read lengths of 80 kb to >1 Mb [16,17], now enable repetitive and structurally complex DNA elements to be resolved with confidence. Nanopore sequencing is particularly useful, as there is no theoretical limit on maximum read length, suggesting that mt genomes can be sequenced outright, irrespective of structural complexities.

The recent discovery of long stretches of tandem-repetitive DNA elements (~4–7 kb) in the mt genomes of parasitic flatworms using advanced sequencing/informatic workflows [18,19,20,21] has emphasised the need to scrutinise published mt genomes of socio-economically important trematodes such as the carcinogenic human blood-fluke, *Schistosoma haematobium*. Although the protein-coding complement of the mt genome of this dioecious trematode had been thoroughly characterised [22], there was an indication that the non-coding region inferred (estimated at 390 bp) was incomplete, being suggestive that structurally complex, repetitive DNA in this region could not be assembled at the time. Here, we used a Nanopore-informatic workflow to tackle this problem. We demonstrate how this workflow resolved this previously-elusive DNA region in the mt genome of *S. haematobium*, and revealed unexpected and marked variability in the length, structure and number of tandem-repeats in this non-coding mt region within this species. This workflow should have broad applicability to elucidating complex non-coding regions in metazoan mt genomes.

## 2. Results

### 2.1. Sequence Data Sets and Mapping Results

**Figure 1:**
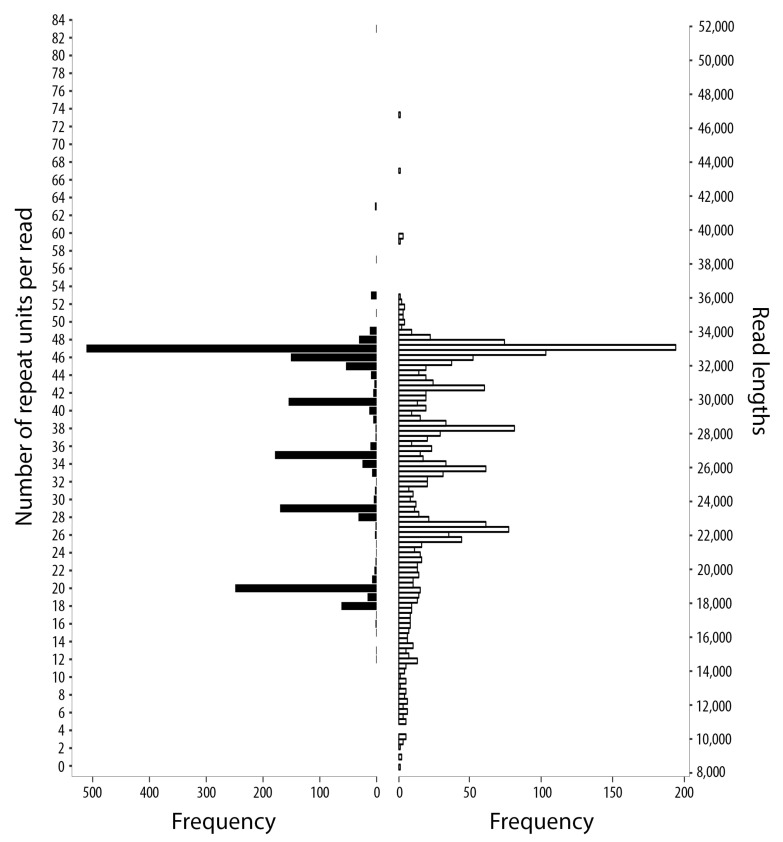
Distribution of repeat unit frequencies and long-read lengths. Both panels represent Nanopore long-reads (*n* = 1760) that spanned the entirety of the tandem-repeat region.

A total of 26,402 long-reads contained sequence tracts that matched the 5′- and 3′-flanking sequences of the incomplete non-coding region of the published mt genome of *S. haematobium* (see [22]). An appraisal of these reads revealed a tandem-repetitive region containing two distinct types of units (designated ShR1 and ShR2). We identified 4098 long-reads containing numerous such units (Figure S1), and 1760 of these reads bridged the entire tandem-repetitive non-coding region (Figure 1 and Figure S1). Then, we examined the nature and extent of repeats within this region.

### 2.2. Complete mt Genomes for S. haematobium, with a Marked Variation in the Number of Repeat Units within the Non-Coding Region

**Figure 2:**
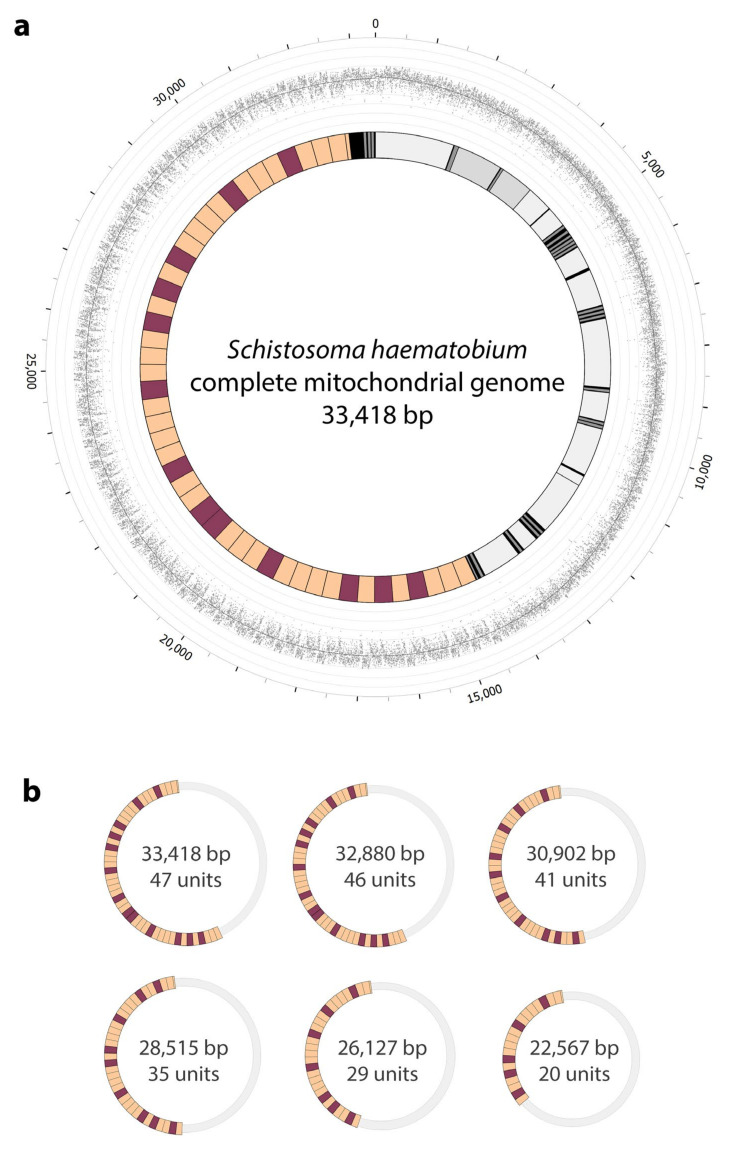
Complete mitochondrial (mt) genomes representing *Schistosoma haematobium*. (**a**) Schematic representation of the dominant, representative mt genome of *S. haematobium* (33,418 bp; inner circle), including the newly-identified tandem-repeat region (18,458 bp; units ShR1 and ShR2 in orange and purple, respectively). The 12 protein-coding genes, 2 rRNAs and 22 tRNAs (in shades of grey) are in accord with a published reference mt genome available in GenBank (accession no. DQ157222; [22]); short non-coding regions (<350 bp) are in black. The outer circle represents the coverage of long-reads (produced by Oxford Nanopore sequencing) across the genome. The graph shows the depth of nucleotides at each position (grey dots) and the smoothed average of depth across the genome (solid dark grey line). Circular axes represent every 100 reads mapped. Numbers on the outer circle represent positions on the genome in base pairs. (**b**) Schematic representation of all established mt genome lengths in *S. haematobium* supported by >150 Nanopore long-reads (cf. Figure 1). Numbers inside represent the length of the mt genome and the number of units in the tandem-repeat region. Sizes of the circles are proportional to genome lengths.

Scrutiny of the 1760 long-reads revealed marked variation in the number of repeat units (range: 12 to 83) within the non-coding region (Figure 1); respective mt genome lengths were estimated at 19.4 and 47.8 kb, and associated non-coding regions were between 4.4 kb and 32.8 kb. Of all long-reads, those with 47, 46, 41, 35, 29 or 20 tandem-repeat units had the highest frequencies (Figure 1; Table 1). These high-frequency reads allowed us to define six complete mt genomes of 33,418 bp to 22,567 bp at high (>550- to 150-times) read-coverage. These representative genomes each harboured a single tandem-repetitive non-coding region varying in size from 18,458 bp to 7607 bp (Table 1; Figure 2 and Figure S2). The accuracy and completeness of the six mt genome sequences were confirmed by read-mapping (Figure 2 and Figure S2), with average nucleotide coverages ranging from 553- to 157-times. An analysis of the 1760 long-reads (range: 8362 to 46,810 bp) revealed that the lengths of the commonest sequences corresponded to those of the representative mt genomes (Figure 1). A comparison of the six mt genomes with that published previously for *S. haematobium* (15,003 bp; GenBank accession no. DQ157222; [22]) revealed a high nucleotide sequence similarity of ≥99.84% (prior to polishing, ≥99.77%) for all 12 protein-coding genes, 22 tRNAs and two rRNAs, and the same gene order.

### 2.3. Structural Features of the Non-Coding Regions

**Figure 3:**
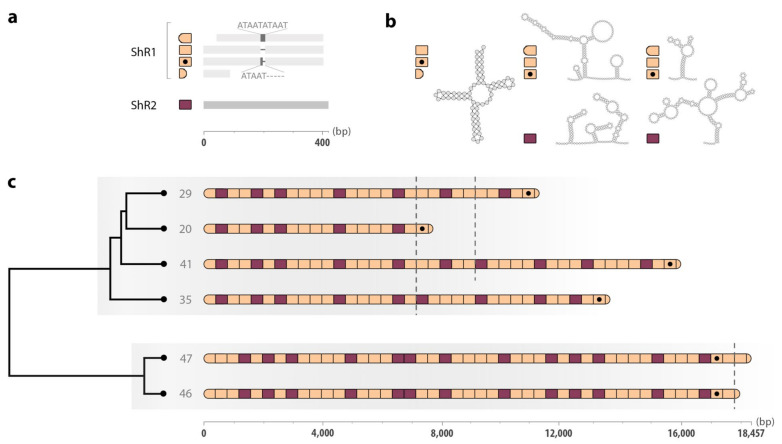
Structure of the tandem-repeat regions in the mitochondrial (mt) genomes of *Schistosoma haematobium*. (**a**) A schematic representation of the units ShR1 (orange) and ShR2 (purple). Insertions of 5′-ATAAT-3′ motifs within a TA-rich region in ShR1s are indicated. Scale in base pairs is shown at the bottom. (**b**) Secondary structures predicted for ShR1 and ShR2 (units in which they occur are indicated on the left of the structure). (**c**) A schematic representation of the hierarchical cluster analysis of repeat unit patterns (cf. Figure S3). Orange and purple shapes correspond to units ShR1 and ShR2 in panels (**a**) and (**b**). Grey numbers at branch tips correspond to the number of units. Lineage 1 (top) and 2 (bottom) are shaded in grey. Dashed lines demarcate the end of common repeat unit patterns within lineages. Scale in base pairs is shown at the bottom.

The annotation of the non-coding region for all six representative mt genomes revealed that units ShR1 (386 to 397 bp) and ShR2 (408 to 420 bp) were repeated in an alternating pattern. However, the first and last units in each tandem-repeat region were incomplete copies of ShR1; such ‘imperfect’ units lacked either the 5′- or 3′-end of ShR1 and were 356 to 358 bp and 90 bp in size, respectively (Figure 3). Irrespective of this size variation, ShR1 units were very similar in sequence upon alignment (average nucleotide identity: >99%; excluding ‘imperfect’ units). The only notable difference occurred in a~70 bp TA-rich sequence tract and related to a 5′-ATAAT-3′ repeat-motif, which was either absent, or repeated once or twice (Figure 3). The ShR2 units in all six genomes were conserved in sequence (average nucleotide identity: 99.30%) upon alignment, with most nucleotide differences relating to single insertion/deletion events (indels) in homopolymers. TA-rich regions in ShR2 units were short and did not exceed 15 bp.

Parts of units ShR1 and ShR2 were predicted to fold into secondary DNA structures, varying from simple hairpin-loops to complex multi-branched structures (Figure 3). Notably, a 65 bp-section at the 5′-ends of ShR1s (excluding the first ShR1 unit of each genome, being incomplete at the 5′-end) assumed a tRNA-like structure (Figure 3) predicted to code for a serine. This sequence was conserved among all such ShR1 units in all six genomes, with a single nucleotide difference detected in only four of a total of 155 units.

### 2.4. Tandem-Repeat Patterns in the Non-Coding Region

The hierarchical cluster analysis of the patterns of units ShR1 and ShR2 in the 1760 long-reads revealed six well-supported groups of reads (with approximately unbiased *p* values at the majority of nodes being ≥95), which corresponded to the non-coding regions of the six mt genomes (Figure 3 and Figure S3). Two dominant lineages of repeat patterns were identified: ‘Lineage 1′ comprising 41, 35, 29 or 20 units, and ‘Lineage 2′ with 47 or 46 units (Figure 3 and Figure S3).

Scrutiny of repeat patterns within each of the two lineages indicated variation in the arrangement of the two units near the 3′-ends of the six distinct non-coding regions (Figure 3). The first and last ‘imperfect’ ShR1 units were consistent in length and nucleotide composition—the first was incomplete at the 5′-end and included a 5′-ATAATATAAT-3′ insertion; the last was consistently a 90 bp tract at the 5′-end. In all tandem-repeat lengths within Lineage 1, the penultimate unit was represented by an ShR1 unit carrying a single 5′-ATAAT-3′ insertion.

## 3. Discussion

The definition of non-coding regions in mt genomes requires an accurate assessment of the lengths, length-frequencies and nucleotide compositions of repetitive sequences. Although this has been challenging to achieve, particularly for expansive tandem-repeat regions and when amount, quality and molecular weight of genomic DNA for analyses are limiting, recent studies using third-generation (long-read) sequencing methods [18,19,20,21,23] have shown considerable promise to overcome this challenge. Here, we have demonstrated the effectiveness of Oxford Nanopore sequencing technology to read through long and complex tandem-repeat regions in mt genomes within a pool of *S. haematobium* adults, and the utility of a practical informatic approach to reliably define consensus non-coding regions and to dissect the nature and extent of heterogeneity within them.

The substantial lengths of the tandem-repetitive regions discovered here were unexpected (Figure 1 and Figure 2). More than a third of each of the six representative mt genomes characterised was repetitive DNA, with the largest genome (33.4 kb) harbouring the longest tandem-repeat region (18.5 kb) recorded to date for a metazoan organism. Usually, non-coding regions exceeded the total length of all protein-coding genes (Table 1; Figure 2). Tandem-repeat regions of <7 kb were rare within the pool of worms sequenced, and we detected one of 32.8 kb with 83 units (Figure 1). Although the biological function of such lengthy tandem-repeat regions is presently unknown, we hypothesise that they contain elements that regulate replication and/or transcription in *S. haematobium*.

In the absence of evolutionary pressure on the copy-number of repeat units, a small mt genome would be expected to have an advantage over a larger variant by being replicated more rapidly [24], leading to an accumulation of small mt genomes in cells. However, there seems to be an active mechanism generating and favouring long mt genome sizes in *S. haematobium*, overriding a putative sized-based selective advantage of a compact genome. Some examples of a selective inheritance of large variants of mt genomes have been attributed to expanded origins of replication, which are thought to increase replication efficiency [25,26,27]. This might be the case for *S. haematobium*. This proposal is plausible because, in other animals (e.g., mammals, birds, fish and insects), repetitive elements, stretches of non-coding DNA, TA-rich regions and/or secondary structures, such as hairpin and clover-leaf conformations (Figure 3), are often present in genuine ‘control’ regions, known to contain regulatory signals for replication and/or transcription [28,29,30,31,32,33]. Such expanded, regulatory regions might be retained solely due to a ‘selfish’ advantage in transmission [26], or may provide a means of ‘fine-tuning’ cellular energy production and genome maintenance strategies to particular environmental conditions, as we have hypothesised previously for other flatworms [20,21].

The six distinct, predominant sizes of tandem-repeat regions discovered suggest that repeat units might be under ‘stabilizing selection’, such that numbers and/or patterns of these units are ‘optimised’ for effective and essential regulation of transcription and replication [34]. Further, in the absence of any selective forces, one would expect the sizes of tandem-repeat regions representing Lineages 1 and 2 (Figure 3) to be randomly distributed. However, the hierarchical cluster analysis conducted here indicated that this was not the case (Figure S3)—there was a clear distinction between individual genomes with 20, 29, 35 or 41 units (exclusive to Lineage 1) and those with 46 or 47 units (Lineage 2), suggesting that the latter lineage might be selectively maintained at a higher and narrower size range. These two lineages might have evolved through sex-specific selection, one being specific to males and the other to females. Sex-specificity would assume a biparental inheritance of mt genomes, which has been suggested for a closely-related species—*S. mansoni* (see [35]). This mode of inheritance might lead to some embryos retaining, and others eliminating male-transmitted mt DNA, resulting in sex-specific mt genomes populating the germline [36]. However, as a pool of worms was sequenced here, we could not definitively establish whether the variation identified in the tandem-repeat region is within or among individual worms, or within or among cells of particular tissues (heteroplasmy). Future work is warranted to obtain long-read data from individual worms (females and males) from distinct, geographically disparate populations of *S. haematobium*, to gain a better appreciation of the diversity in length and structure of the tandem-repeat regions in the mt genome of this species, and to attempt to establish the selection pressure(s) leading to such variation.

The diversity in the length and composition of repeat units in mt tandem-repetitive, non-coding regions in *S. haematobium* raises a question as to the molecular mechanisms underlying this variation in flatworms. Lineage-specific variation in repeat patterns indicates that mechanisms leading to unit expansions, contractions and/or rearrangements appear to conserve the order of these units at the 5′-ends of tandem-repeat regions, whereas modifications seem to occur at the 3′-ends (Figure 3). Although the mutational processes leading to this diversity are unknown in flatworms, slipped-strand mispairing, imprecise or pre-mature termination of replication and/or inter- or intra-molecular recombination events during genome replication and/or maintenance have been proposed [8,37,38,39,40,41]. Clearly, these aspects warrant investigation. Future work might utilise two-dimensional neutral agarose gel electrophoresis and electron microscopy techniques [42] to explore the mode(s) of replication in *S. haematobium*, and to investigate whether tandem-repeat regions contain replication origins.

The presence of expansive tandem-repetitive regions is likely a common feature of schistosome species, as repetitive sequence tracts of ≥4 kb have been identified in *S. bovis* (partial assembly of PacBio-based long-read data; [19]), and detected in *S. mansoni, S. japonicum* and *S. mekongi* (short-read data, or restriction fragment length polymorphism and Southern blotting results; [11,43,44,45]). In other metazoans, long mt tandem-repetitive regions (>3 kb) seem to be rare, yet have been indicated in some phylogenetically divergent animal lineages including nematodes [10], insects [9,46], birds [47] and amphibians [23]. The propensity of some animal species/lineages to generate and tolerate lengthy tandem-repeat regions, while others select for a relatively short length is puzzling, and the evolutionary drive underlying such distinct architectures remains to be systematically addressed. Such investigations have largely been hampered by the inability of conventional methods to sequence across complex repetitive, non-coding regions, which has led to gaps in many published mt genome assemblies [48,49,50,51,52,53]. Clearly, Nanopore technology, with its ability to sequence long, intact DNA strands, without the need for read-assembly, lends itself well to the decoding of the mt genomes of a wide range of taxa across the Tree of Life [54]. Such an effort could open up new areas of investigation into the function and evolution of non-coding regions in the mt genomes of eukaryotes.

## 4. Materials and Methods

### 4.1. Parasite Material

An Egyptian strain of *S. haematobium*, for which a draft nuclear genome has been characterised [55,56], was used here. This strain is maintained in the Biomedical Research Institute, Rockville, Maryland [57] in *Bulinus truncatus* (intermediate snail host) and *Mesocricetus auratus* (hamster; mammalian definitive host). Adult worms were prepared and stored as described previously [55].

### 4.2. Isolation of High Molecular Weight Genomic DNA, Library Construction and Sequencing

High-quality total genomic DNA was isolated from a pool of 50 male and female adult worm pairs of *S. haematobium* using the Circulomics Tissue Kit (Circulomics, Baltimore, MD, USA) and used to construct two rapid-sequencing (SQK-RAD004) and two ligation-sequencing genomic DNA libraries (SQK-LSK109), according to the manufacturer’s protocol (Oxford Nanopore Technologies, Oxford, UK). For one rapid-sequencing and one ligation-sequencing library, low molecular weight DNA was removed using the 10 kb-Short Read Eliminator (SRE) kit (Circulomics, Baltimore, MD, USA). Each library was sequenced (48 h) in a distinct flow cell (R9.4.1) using the MinION sequencer (Oxford Nanopore Technologies). Following sequencing, bases were ‘called’ from HDF5 files (FAST5 format) using the program Guppy v.3.1.5 (Oxford Nanopore Technologies) and stored in the FASTQ format [58].

### 4.3. Defining the mt Genomes

First, long-reads containing sequence tracts that matched perfectly those flanking the incomplete non-coding region (i.e., positions 4921 to 5420 at the 5′-end and 5465 to 5964 at the 3′-end) of the published mt genome of *S. haematobium* from Mali (GenBank accession no. DQ157222; [22]) were identified using the BLASTn tool [59]. These reads were then assembled using the program Canu v. 2.0 [60], and repeats identified using the program ‘repeat-match’ in the MUMmer package v. 3.23 [61]. A library of identified repeat units and published mt protein genes of *S. haematobium* (cf. DQ157222; [22]) was used to critically assess the completeness of the non-coding region and the frequency of such units in reads identified using the program RepeatMasker v. 4.0.5 (http://www.repeatmasker.org). As some sequence reads produced using Nanopore technology can contain random errors, only reads with high coverage (≥150-times) with the commonest repeat unit frequencies (±1 unit, with no overlap permitted) were used to define consensus sequences. Coding regions were then polished with available short-read data [55] using Pilon v. 1.23 [62]. Finally, long-read data were mapped to the defined mt genomes using Minimap2 [63] (alignment threshold: 70% of read length), and coverage of the genomes was determined using mpileup in the SAMtools package [64]. The frequency of repeat units in long-reads, read lengths and nucleotide coverages were plotted using the software package R [65]; circular plots were generated using the tool Circos v0.69-8 [66].

### 4.4. Annotation of the mt Genomes and Characterisation of the Tandem-Repeat Regions

The newly-defined mt genomes were compared with that published for *S. haematobium* (DQ157222; [22]), and tRNA, rRNA and protein-coding genes annotated accordingly. The open reading frame (ORF) of each protein-coding gene was verified using the program Geneious v.11.1.5 [67], employing the mt genetic code for echinoderms and flatworms ([68]; https://www.ncbi.nlm.nih.gov/Taxonomy/Utils/wprintgc.cgi#SG9). Secondary structures were predicted using the Vienna RNA Websuite [69] and drawn using the tool Forna [70]. Complete mt genome sequences were deposited in the GenBank database under the accession nos. MW067222—MW067227; raw data are available in the Sequence Read Archive (SRA) under the accession no. PRJNA78265.

### 4.5. Hierarchical Cluster Analysis

The repeat units and their order in long-reads spanning the entirety of the non-coding region were established using the program RepeatMasker v. 4.0.5, employing a library of identified units. For each long-read, the units within this region were used to create a document-term matrix employing the textmineR v. 3.0.4 package in R (www.rtextminer.com). This matrix was then re-weighted using the TF-IDF method by multiplying the repeat term frequency (TF) by an inverse document frequency (IDF) using textmineR. Subsequently, the reweighted matrix was subjected to hierarchical clustering using pvclust v. 2.2-0 [71], and using a correlation distance measure, the Ward’s (ward.D) agglomerative clustering method and 10,000 bootstrap replicates. The final dendrogram plot was created using the ggplot2 package v. 3.3.2 in R.

## Figures and Tables

**Table 1:** Features of the six representative mitochondrial (mt) genomes defined for *Schistosoma haematobium* and their respective tandem-repetitive, non-coding regions.

Features (cf. Figure 2, Panel b)						
Mt genome length/size (bp)	33,418	32,880	30,902	28,515	26,127	22,567
Length of tandem-repeat region (bp)	18,458	17,920	15,942	13,555	11,167	7607
Number of repeat units in this non-coding region Tandem-repeat region relative to mt genome length/size (%) No. of long-reads representing the tandem-repeat region	47 55.2 511	46 54.5 151	41 51.6 155	35 47.5 179	29 42.7 170	20 33.7 249

## Data Availability

Complete mitochondrial genome sequences have been deposited in the GenBank database under the accession nos. MW067222—MW067227; raw data are available in the Sequence Read Archive (SRA) under the accession no. PRJNA78265.

## Supplementary Materials

The following are available online at https://www.mdpi.com/1422-0067/22/4/1811/s1, Figure S1: distribution of repeat unit frequencies in Nanopore long-reads, Figure S2: nucleotide coverage of Nanopore long-reads across mitochondrial (mt) genomes with 46, 41, 35, 29 and 20 repeat units, Figure S3: hierarchical cluster analysis of the patterns of repeat units in the mitochondrial (mt) genomes of *Schistosoma haematobium*.
