# Supplementary material for: Nanopore Sequencing Resolves Elusive Long Tandem-Repeat Regions in Mitochondrial Genomes
Source: Int J Mol Sci. 2021 Feb 11;22(4):1811. doi: 10.3390/ijms22041811 (PMC7918261; doi:10.3390/ijms22041811)
Supplement: Supplementary file 1 [file ijms-22-01811-s001.pdf]

International Journal of
Molecular Sciences

Supplementary Materials:

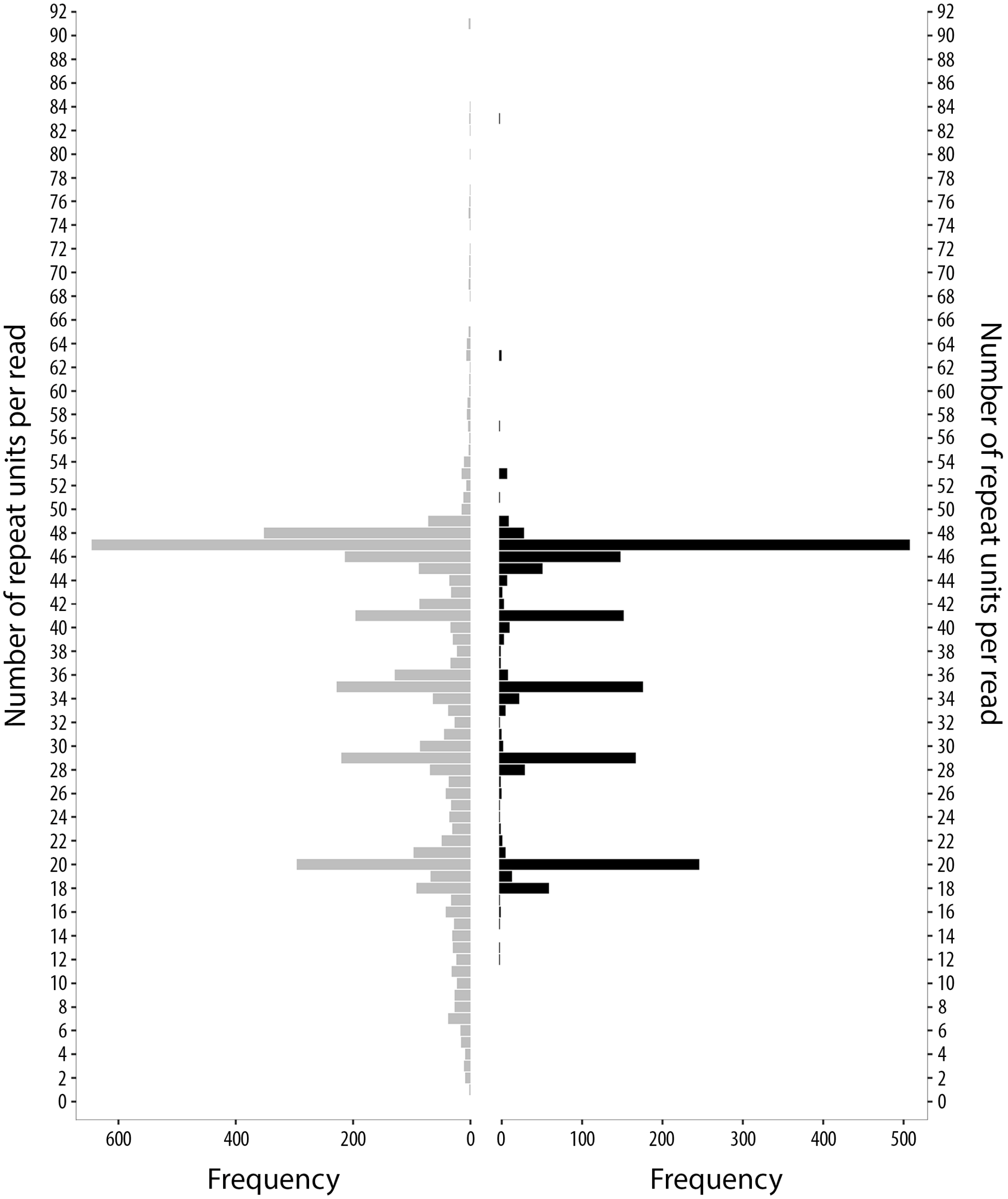

Figure S1. Distribution of repeat unit frequencies in Nanopore long-reads. The left-side panel represents repeat unit frequencies in all reads (*n* = 4098) containing the sequences of repeat units ShR1 and ShR2. The right-side panel represents repeat unit frequencies in a subset of reads (*n* = 1760) that spanned the entirety of the tandem-repeat region.

International Journal of
Molecular Sciences

MDPI

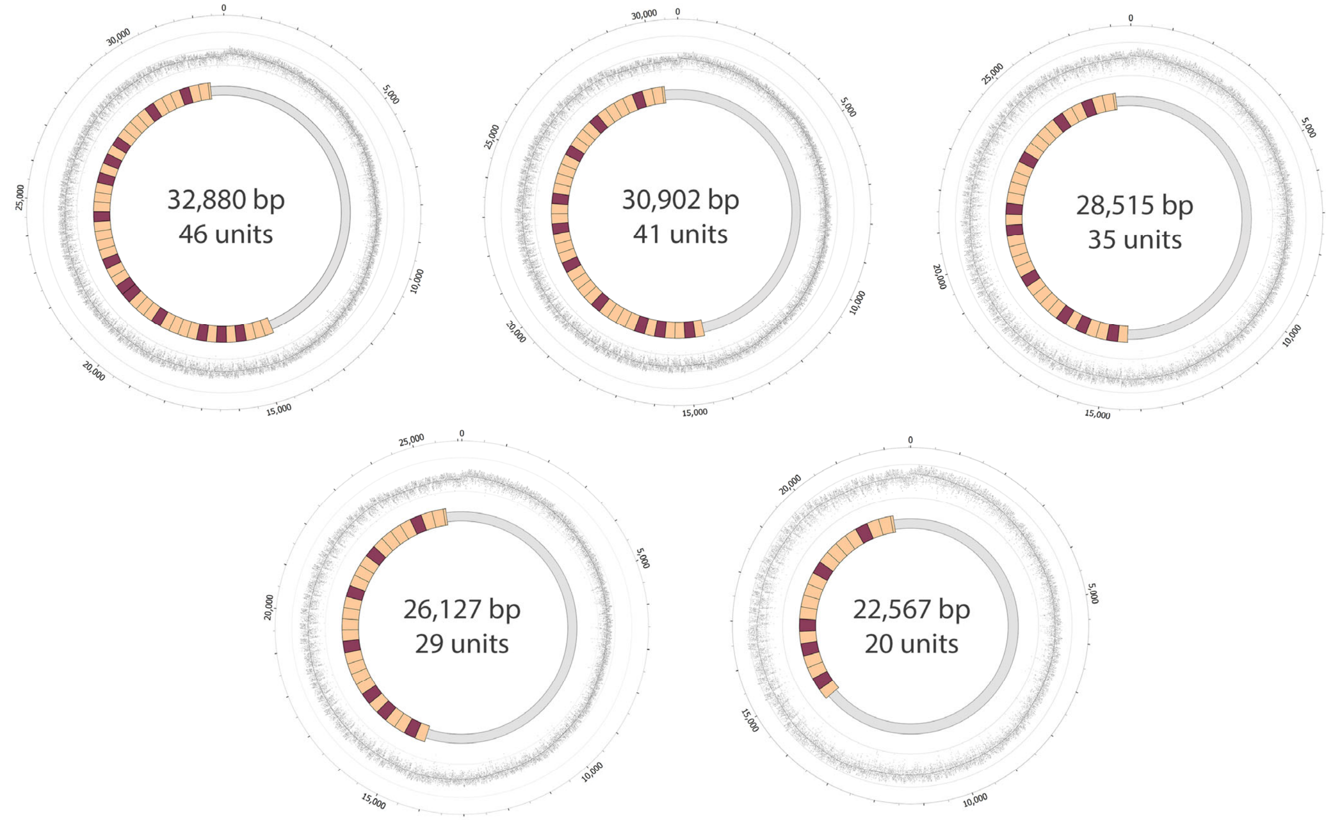

Figure S2. Nucleotide coverage of Nanopore long-reads across mitochondrial (mt) genomes with 46, 41, 35, 29 and 20 repeat units. Tandem-repeat regions are in orange (repeat unit ShR1) and purple (ShR2). The outer circles represent the coverage of long-reads across the genomes. Each graph shows the depth of nucleotides at each position (grey dots) and the smoothed average of depth across the genomes (solid dark grey lines). Circular axes represent every 100 reads mapped. Numbers on the outer circle represent positions on the genomes in base pairs. Numbers inside represent the length of the mt genome and the number of units in the tandem-repeat region. Sizes of the circles are proportional to genome lengths.

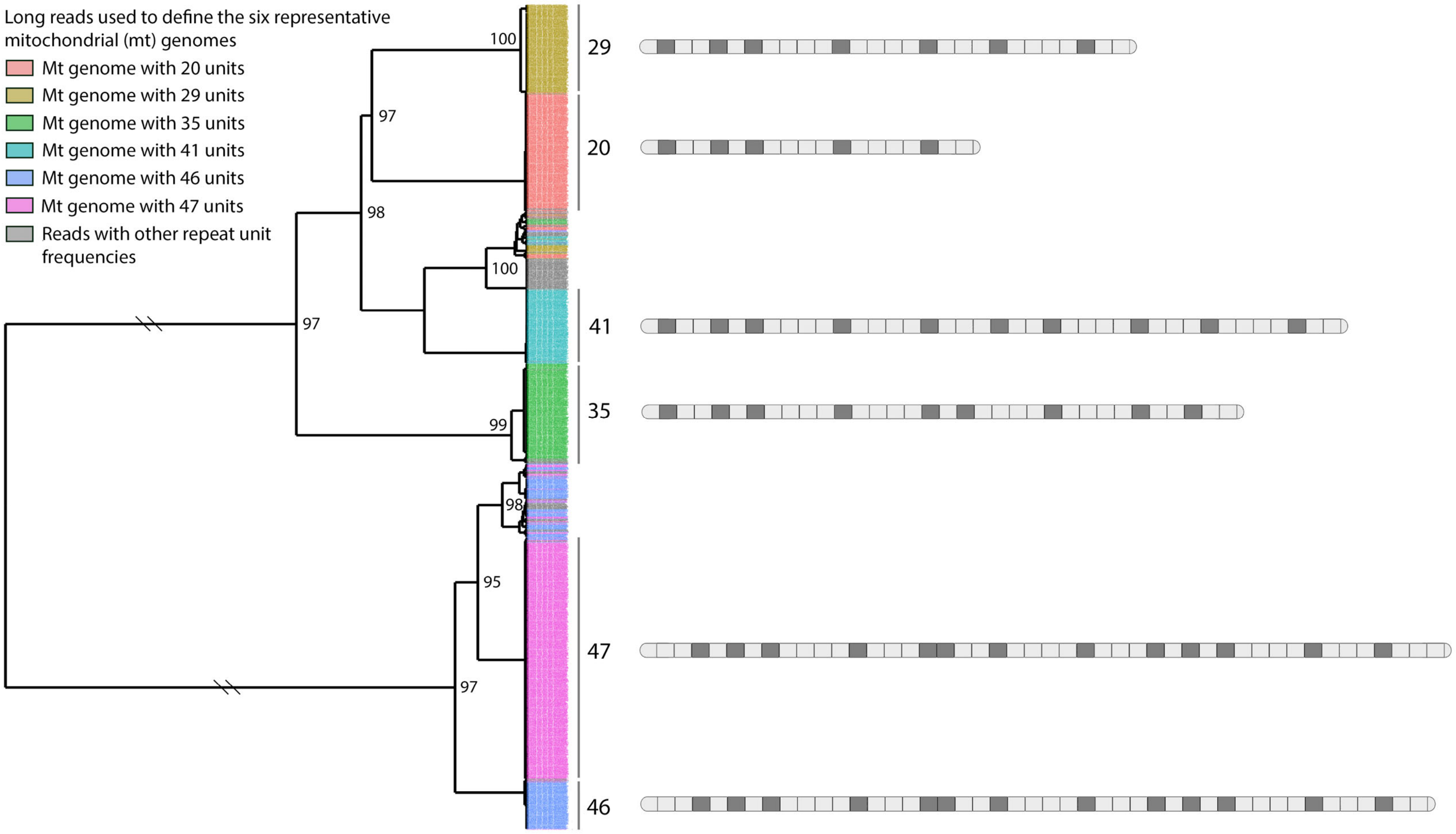

Figure S3. Hierarchical cluster analysis of the patterns of repeat units in the mitochondrial (mt) genomes of *Schistosoma haematobium*. All reads represent Nanopore long-reads (n = 1760) that spanned the entirety of the tandem-repeat region. Numbers at the nodes represent approximately unbiased p values (values >95 are shown). Individual reads (coloured) are at branch tips; colours correspond to different genomes (see figure legend). Clusters of reads are indicated with vertical lines. Light and dark grey shapes correspond to units ShR1 and ShR2, respectively; numbers (left) represent the number of units. .
